# Supplementary material for: Sphingosine d18:1 promotes nonalcoholic steatohepatitis by inhibiting macrophage HIF-2α
Source: Nat Commun. 2024 Jun 4;15:4755. doi: 10.1038/s41467-024-48954-2 (PMC11150497; doi:10.1038/s41467-024-48954-2)
Supplement: Supplementary file 1 — Supplementary Information [file 41467_2024_48954_MOESM1_ESM.pdf]

Sphingosine d18:1 Promotes Nonalcoholic Steatohepatitis by Inhibiting Macrophage HIF-2α

Jialin Xia[1,2,3,4,7,11], Hong Chen[1,2,3,4,7,11], Xiaoxiao Wang[9,11], Weixuan Chen[4,7], Jun Lin[1,2,3], Feng Xu[1,2,3], Qixing Nie[1,2,3], Chuan Ye[1,2,3], Bitao Zhong[1], Min Zhao[4,7], Chuyu Yun[4,7], Guangyi Zeng[1,2,3,4,7], Yuejian Mao[5], Yongping Wen[5], Xuguang Zhang[5,6], Sen Yan[4,7], Xuemei Wang[1,2,3], Lulu Sun[7,8], Feng Liu[9], Chao Zhong[10], Pengyan Xia[10], Changtao Jiang[1,2,3,10], Huiying Rao[9*], and Yanli Pang[1,4,7*]

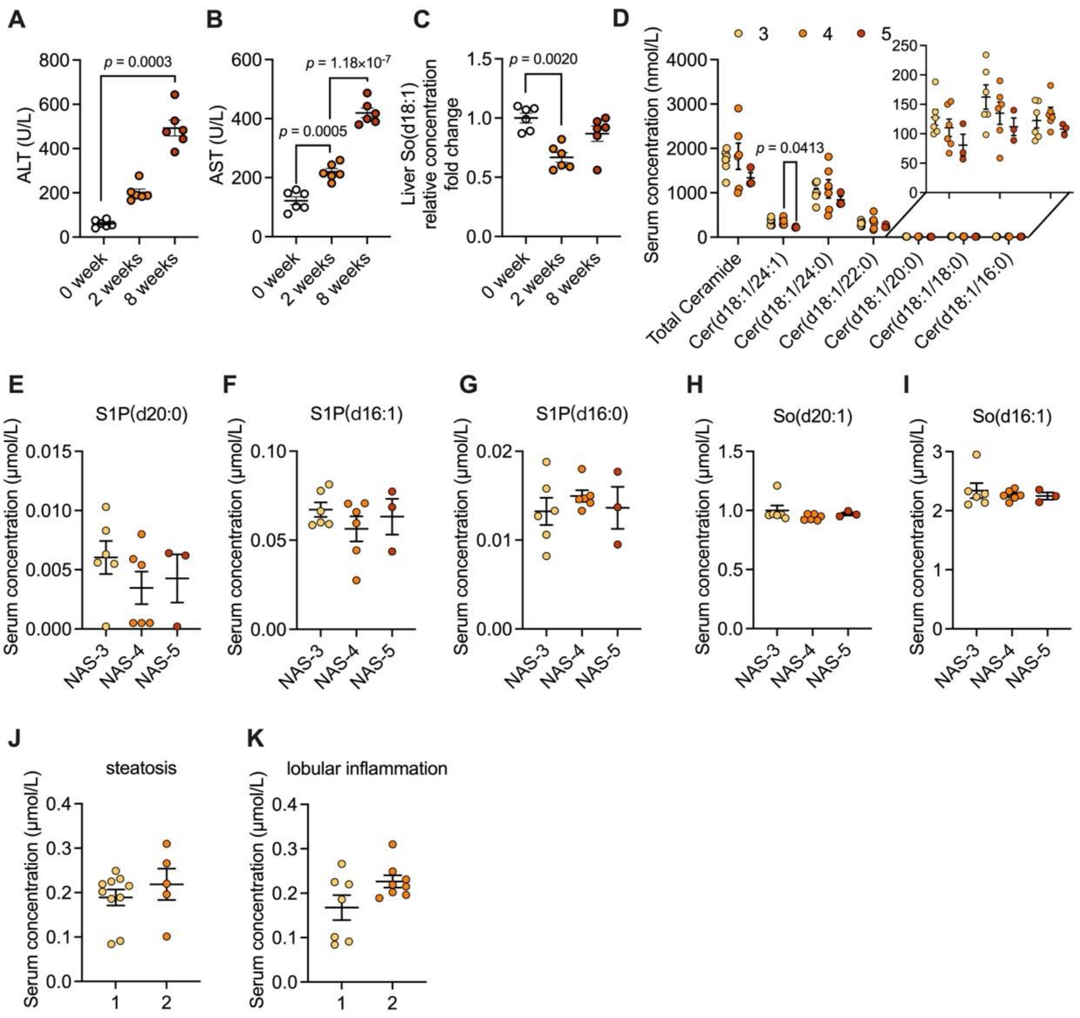

Supplementary Figures

Supplementary Figure 1 Serum concentration of sphingolipid in NASH patients

A-C, serum ALT (A), AST (B) and liver So(d18:1) relative concentration fold changes (C) in different time point of NASH modeling mice. D-I, levels of ceramides (D), S1Ps (E-G) and other types of sphingosines (H-I) in the serum of patients in different NAFLD stages classified by NAS scores. J, K, serum So(d18:1) concentration of patients in different steatosis stages (J) and different lobular inflammation stages (K). Patients with NAS $\geq$ 5 were considered definite NASH, patients with scores of 3 or 4 were considered borderline NASH, and patients with scores of less than 3 were diagnosed as NAFL.

Data are the means ± s.e.m., K, statistical analysis was performed using two-tailed Student's t-tests; J, statistical analysis were performed using two-tailed Mann-Whitney U-tests. B, D, E-G, statistical analysis were performed using One-way ANOVA. A, C, Total Ceramide, Cer(d18:1/24:0) in D, H and I, statistical analysis were performed using Kruskal-Wallis test with Dunn's test.

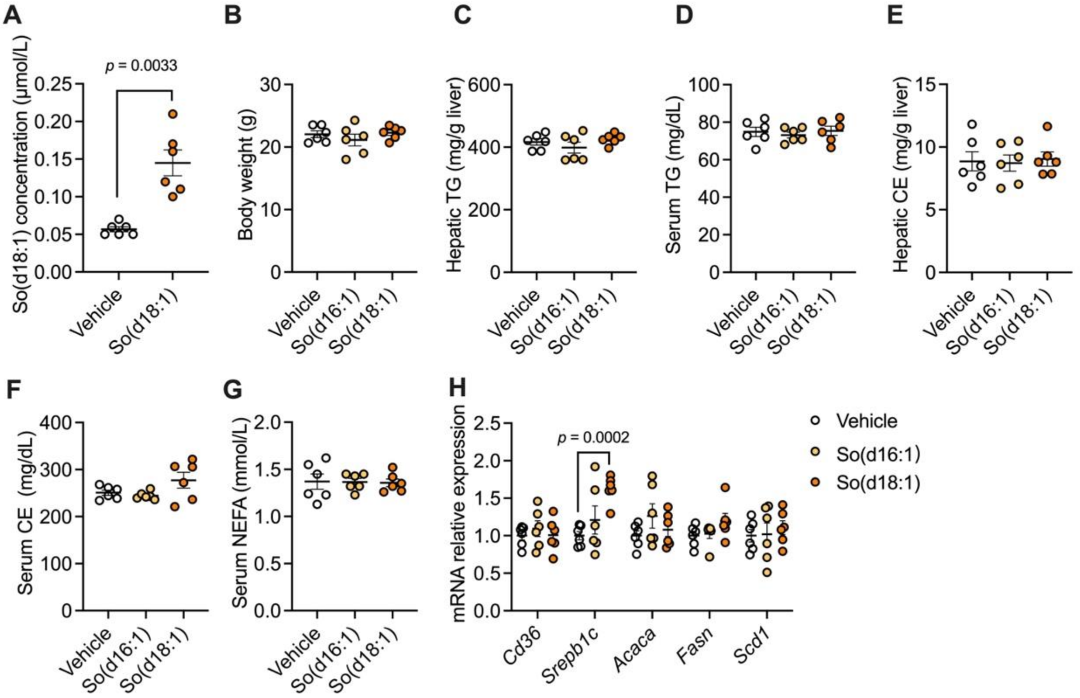

Supplementary Figure 2 So(d18:1) could not influence the lipid metabolism in NASH progression

CDAA-HFD-fed mice were treated with vehicle, sphingosine 16:1 or sphingosine 18:1 for 8 weeks (n=6 mice/group). A, serum So(d18:1) concentration of mice injected with vehicle or sphingosine 18:1. B, body weights. C, hepatic TG. D, serum TG. E, hepatic CE. F, serum CE. G, serum NEFA. H, relative mRNA levels of genes related to lipid metabolism.

Data are the means ± s.e.m. A, statistical analysis was performed using two-tailed Student's t-tests; B-G, statistical analysis was performed using One-way ANOVA; *Fasn* in G, statistical analysis was performed using the Kruskal-Wallis test.

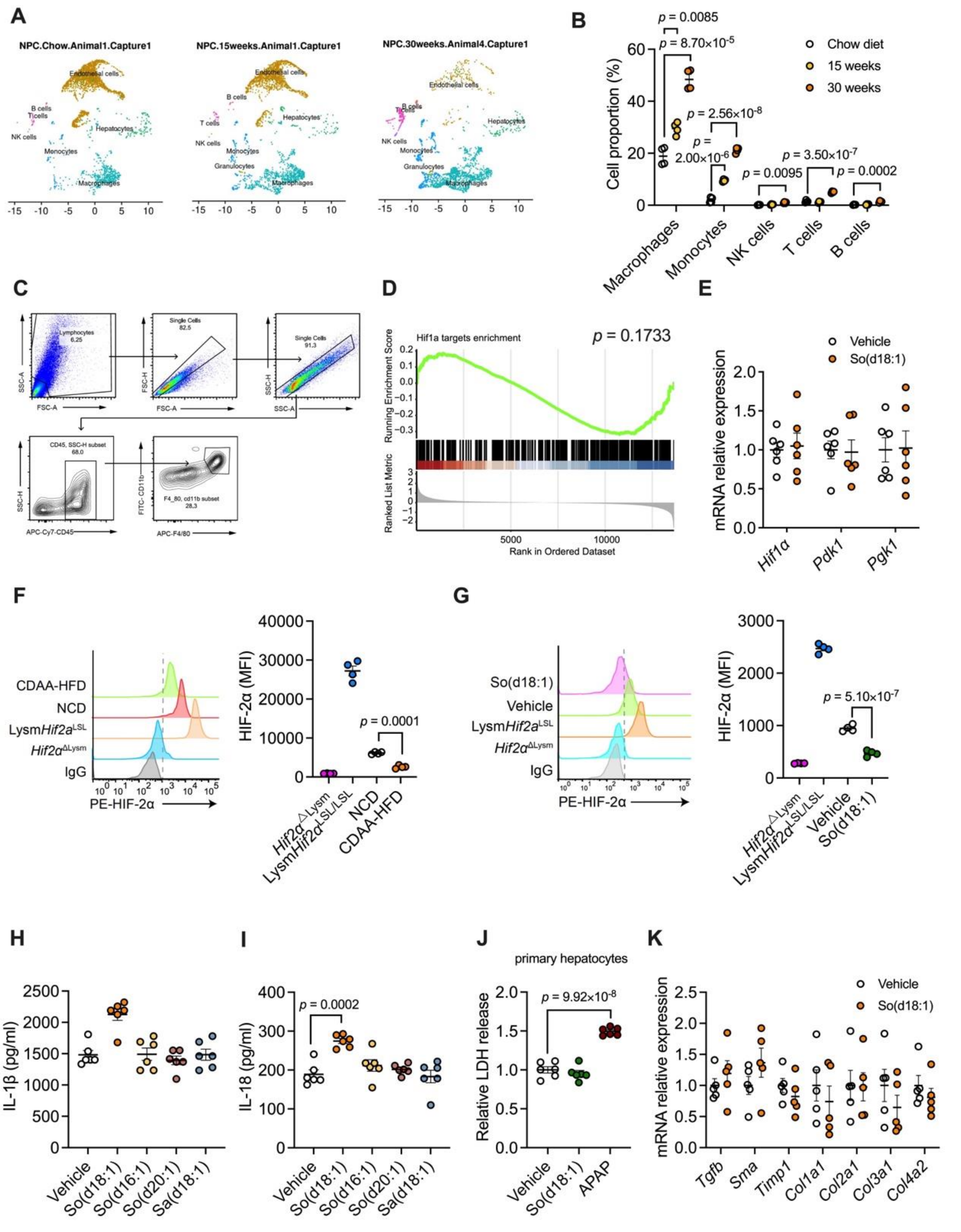

Supplementary Figure 3 So(d18:1) inhibits HIF-2α transcription function in liver macrophages

A and B, analyze of non-parenchymal cells subcluster single-cell RNA-sequencing data from mice fed with chow diet or HFHFD diet for 15 weeks or 30 weeks. Cell clustering changed through NASH progression (A), and macrophages and monocytes increased largely (B). (n=6). C, gating strategy of liver macrophages which were characterized as live $CD45^{+}F4/80^{+}CD11b^{+}$. D, *Hif1a* targets enrichment had no change after So(d18:1) treatment. (n=4). E, relative mRNA levels of *Hif1α* and its downstream target genes in

macrophages treated with vehicle or So(d18:1). (n=6). F, flow cytometric and statistical analysis of HIF-2a in liver macrophages from chow diet or CDAA-HFD diet fed wild-type mice. (n = 4 per group). The signal of HIF2a of BMDMs isolated from LysMHif2α$^{LSL/LSL}$ and Hif2α$^{\triangle LysM}$ BMDMs were used as positive and negative control. G, flow cytometric and statistical analysis of HIF-2a in liver macrophages from CDAA-HFD diet fed mice treated with vehicle (5% CMC-Na) and So(d18:1). (n = 4 per group). The signal of HIF2a of BMDMs isolated from LysMHif2α$^{LSL/LSL}$ and Hif2α$^{\triangle LysM}$ BMDMs were used as positive and negative control. H and I, protein level of IL-1β (H), IL-18 (I) from BMDMs treated with vehicle, So(d18:1), So(d16:1), So(d20:1) or Sa(d18:1) under NLRP3 inflammasome stimulation. (n=6). J, Cell viability was determined by LDH release. Primary hepatocytes were treated with vehicle, So(d18:1) (20 μM) or APAP (5 mM) for 24 h. n = 5. K, relative mRNA levels of genes related to fibrosis in LX-2 cells treated with vehicle or So(d18:1). (n=6).

Data are the means ± s.e.m. B, F, G, I, J, statistical analysis was performed using One-way ANOVA. E, K, statistical analysis was performed using two-tailed Student's t-tests. H, statistical analysis was performed using the Kruskal-Wallis test.

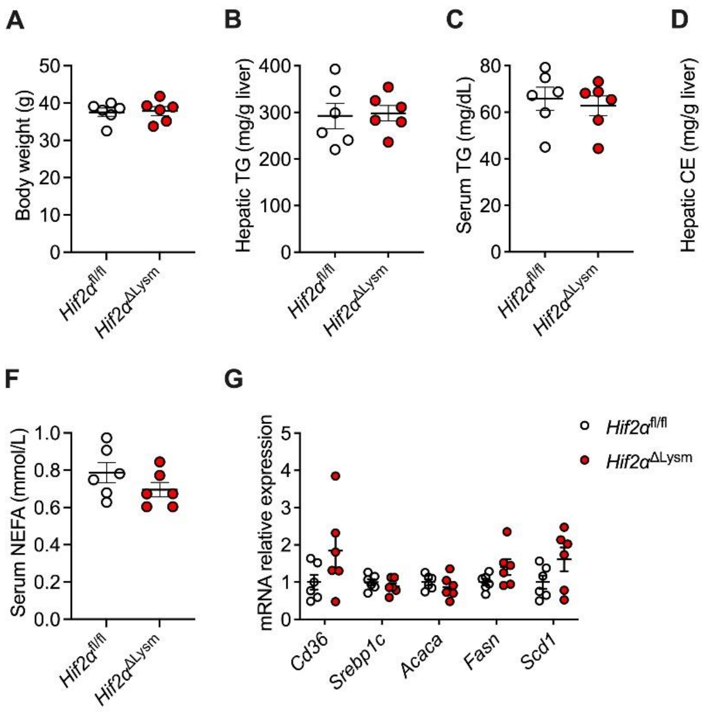

Supplementary Figure 4 HIF-2α KO in macrophages didn't affect lipid metabolism in liver.

Eight-week-old male *Hif2α*$^{\mathrm{fl/fl}}$ and *Hif2α*$^{\Delta\mathrm{Lysm}}$ mice were administered a GAN diet for 24 weeks (SPF, n=6 mice/group). A, body weights. B, hepatic TG. C, serum TG. D, hepatic CE. E, serum CE. F, serum NEFA. G, relative mRNA levels of genes related to lipid metabolism.

Data are the means ± s.e.m. A-G, statistical analysis was performed using two-tailed Student's t-tests.

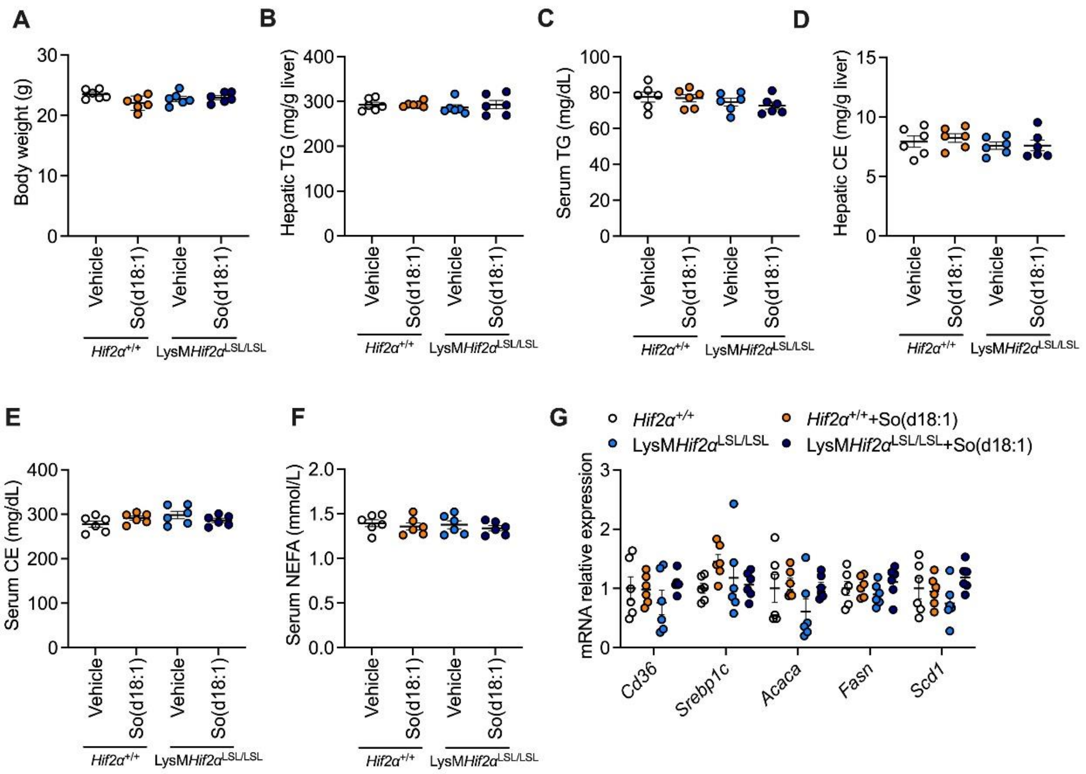

Supplementary Figure 5 HIF-2α overexpression in macrophages didn't affect lipid metabolism

Eight-week-old male *Hif2α*$^{+/+}$ and LysM*Hif2α*$^{LSL/LSL}$ mice were treated with or without So(d18:1) by daily intraperitoneal injection under CDAA-HFD for 8 weeks (SPF, n=6 mice/group). A, body weights. B, hepatic TG. C, serum TG. D, hepatic CE. E, serum CE. F, serum NEFA. G, relative mRNA levels of genes related to lipid metabolism.

Data are the means ± s.e.m. A, C-G, statistical analysis was performed using one-way ANOVA. B, statistical analysis was performed using the Kruskal-Wallis test.

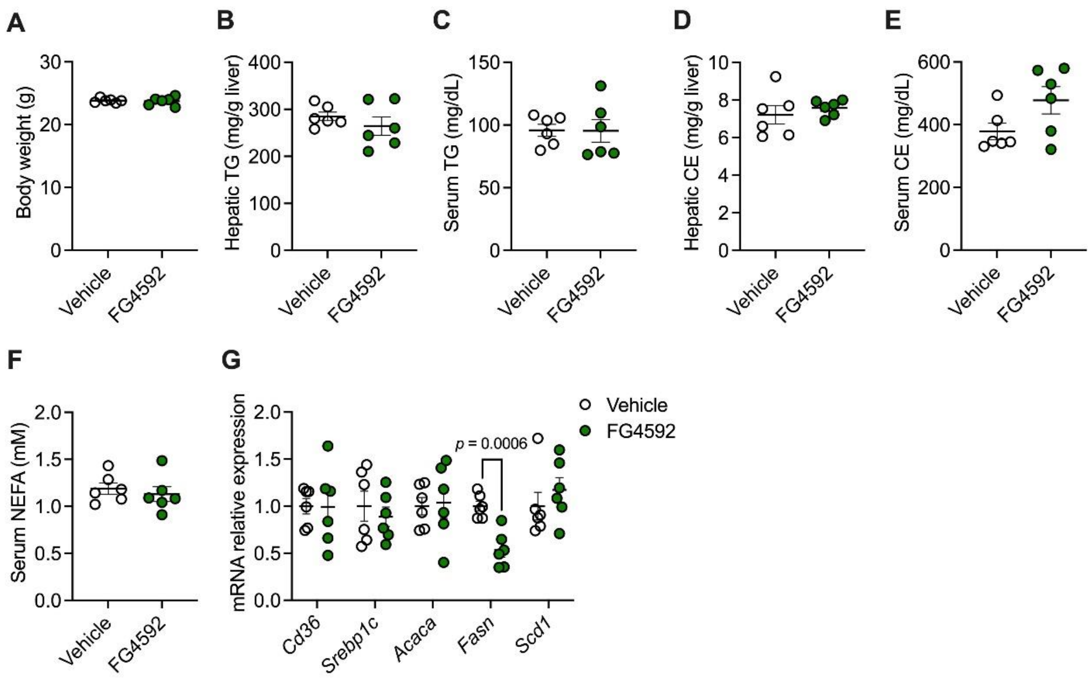

Supplementary Figure 6 FG-4592 didn't affect lipid metabolism

CDAA-HFD-fed mice were treated with vehicle or FG-4592 for 8 weeks (n=6 mice/group). A, body weights. B, hepatic TG. C, serum TG. D, hepatic CE. E, serum CE. F, serum NEFA. G, relative mRNA levels of genes related to lipid metabolism.

Data are the means ± s.e.m. A-D, F-G, statistical analysis was performed using two-tailed Student's t-tests. E, *Scd1* in G, statistical analysis was performed using two-tailed Mann-Whitney U-tests.

Supplementary Table 1 Demographic characteristics of the subjects in clinical patient cohort.

	Healthy volunteer	NASH patient	p value
Female sex (%)	50	50	
Age (y)	45.63 ± 14.97	43.00 ± 14.53	0.6184
BMI (kg/m^2)	21.68 ± 1.94	27.64 ± 5.14	0.0003
TG (mmol/L)	0.98 ± 0.36	1.66 ± 0.59	0.0006
TC (mmol/L)	4.91 ± 1.06	5.07 ± 1.19	0.6849
ALT (U/L)	16.44 ± 5.92	106.63 ± 56.80	$1.25*10^{-5}$
GGT (U/L)	22.31 ± 11.38	91.44 ± 79.70	$3.08*10^{-5}$
AST (U/L)	21.06 ± 5.30	66.38 ± 20.34	$1.37*10^{-6}$
Fibroscan (CAP)	180.88 ± 43.14	274.88 ± 73.10	0.0002

This table shows the general information of healthy volunteers and NASH patients. There ages and gender are totally matched. The data were shown as mean ± SD. Age (y), BMI (kg/m^2), TG (mmol/L), TC (mmol/L), ALT (U/L) p=0.00001249, statistical analysis was performed using two-tailed Student's t-tests. GGT (U/L), p=0.00003082, AST (U/L), p=$1.3718*10^{-6}$, Fibroscan (CAP), statistical analysis was performed two-sided using Mann-Whitney U-tests.

Supplementary Table 2 RT-qPCR primers

Genes	Primer sequences
Hif2a Fwd	5′- CTGAGGAAGGAGAAATCCCGT-3′
Hif2a Rev	5′- TGTGTCCGAAGGAAGCTGATG-3′
Arg1 Fwd	5′-AAGAATGGAAGAGTCAGTGTGG-3′
Arg1 Rev	5′-GGGAGTGTTGATGTCAGTGTG-3′
VEGF Fwd	5′- GGAGATCCTTCGAGGAGCACTT-3′
VEGF Rev	5′- GGCGATTTAGCAGCAGATATAAGAA -3′
Spint Fwd	5′-GTCGGCGTATGGCTCCTT -3′
Spint Rev	5′-GCTTCGGTGTCCAGCACAA-3′
Depdc7 Fwd	5′-AGCAGAGCTCCTGGTAAATGG-3′
Depdc7 Rev	5′-AGCCGTCTAAACTCCTCCCT-3′
Il10 Fwd	5′-ACCTGCTCCACTGCCTTGCT-3′
Il10 Rev	5′-GGTTGCCAAGCCTTATCGGA-3′
Tnfa Fwd	5′-AGGGTCTGGGCCATAGAACT-3′
Tnfa Rev	5′-CCACCACGCTCTTCTGTCTAC-3′
Il1b Fwd	5′-AAGAGCTTCAGGCAGGCAGTATCA-3′
Il1b Rev	5′-TGCAGCTGTCTAGGAACGTCA-3′
Il6 Fwd	5′-TAGTCCTTCCTACCCCAATTTCC-3′
Il6 Rev	5′-TTGGTCCTTAGCCACTCCTTC-3′
Ccl2 Fwd	5′-TTAAAAACCTGGATCGGAACCAA-3′
Ccl2 Rev	5′-GCATTAGCTTCAGATTTACGGGT-3′
F4/80 Fwd	5′-GGATGTACAGATGGGGGATG-3′
F4/80 Rev	5′-CATAAGCTGGGCAAGTGGTA-3′
Tgfb Fwd	5′-GTCACTGGAGTTGTACGGCA-3′
Tgfb Rev	5′-GGGCTGATCCCGTTGATTTC-3′
Sma Fwd	5′-CCAGCCATCTTTCATTGGGATG-3′
Sma Rev	5′-TACCCCCTGACAGGACGTTG-3′
Timp1 Fwd	5′-CCTTTGCATCTCTGGCATCT-3′
Timp1 Rev	5′-CTCGTTGATTTCTGGGGAAC-3′
Col1a1 Fwd	5′-TAGGCCATTGTGTATGCAGC-3′
Col1a1 Rev	5′-ACATGTTCAGCTTTGTGGACC-3′
Col2a1 Fwd	5′-TGAGGTCTGGGTAAAGGCAA-3′

Col2a1 Rev	5′-GTATGAGGTCACCGTCCAGG-3′
Col3a1 Fwd	5′-TAGGACTGACCAAGGTGGCT-3′
Col3a1 Rev	5′-GGAACCTGGTTTCTTCTCACC-3′
Col4a1 Fwd	5′-CACATTTTCCACAGCCAGAG-3′
Col4a1 Rev	5′-GTCTGGCTTCTGCTGCTCTT-3′
Col4a2 Fwd	5′- GCCCTGTAGTCCTGGGAATC -3′
Col4a2 Rev	5′- CCAGTGCTACCCGGAGAAA -3′
Col5a2 Fwd	5′- CATGGAGAAGGTTTCCAAATG -3′
Col5a2 Rev	5′- AAAGCCCAGGAACAAGAGAA -3′
Cd36 Fwd	5′-AGATGACGTGGCAAAGAACAG-3′
Cd36 Rev	5′-CCTTGGCTAGATAACGAACTCTG-3′
Srebp1c Fwd	5′-GGAGCCATGGATTGCACATT-3′
Srebp1c Rev	5′-GCTTCCAGAGAGGAGGCCAG-3′
Acaca Fwd	5′-ATGGGCGGAATGGTCTCTTTC-3′
Acaca Rev	5′-TGGGGACCTTGTCTTCATCAT-3′
Fasn Fwd	5′-GGAGGTGGTGATAGCCGGTAT-3′
Fasn Rev	5′-TGGGTAATCCATAGAGCCCAG- 3′
Scd1 Fwd	5′-TTCTTGCGATACACTCTGGTGC-3′
Scd1 Rev	5′-CGGGATTGAATGTTCTTGTCGT-3′
β-Actin Fwd	5′-GGCTGTATTCCCCTCCATCG-3′
β-Actin Rev	5′-CCAGTTGGTAACAATGCCATGT-3′
