# Supplementary material for: Sphingosine d18:1 promotes nonalcoholic steatohepatitis by inhibiting macrophage HIF-2α
Source: Nat Commun. 2024 Jun 4;15:4755. doi: 10.1038/s41467-024-48954-2 (PMC11150497; doi:10.1038/s41467-024-48954-2)
Supplement: Supplementary file 3 — Reporting Summary [file 41467_2024_48954_MOESM3_ESM.pdf]

nature portfolio

Corresponding author(s): Yanli Pang, Changtao Jiang, Huiying Rao

Last updated by author(s): Mar 22, 2024

Reporting Summary

Nature Portfolio wishes to improve the reproducibility of the work that we publish. This form provides structure for consistency and transparency in reporting. For further information on Nature Portfolio policies, see our Editorial Policies and the Editorial Policy Checklist.

Statistics

For all statistical analyses, confirm that the following items are present in the figure legend, table legend, main text, or Methods section.

n/a	Confirmed	
☐	☒	The exact sample size (*n*) for each experimental group/condition, given as a discrete number and unit of measurement
☐	☒	A statement on whether measurements were taken from distinct samples or whether the same sample was measured repeatedly
☐	☒	The statistical test(s) used AND whether they are one- or two-sided<br>*Only common tests should be described solely by name; describe more complex techniques in the Methods section.*
☐	☒	A description of all covariates tested
☐	☒	A description of any assumptions or corrections, such as tests of normality and adjustment for multiple comparisons
☐	☒	A full description of the statistical parameters including central tendency (e.g. means) or other basic estimates (e.g. regression coefficient) AND variation (e.g. standard deviation) or associated estimates of uncertainty (e.g. confidence intervals)
☐	☒	For null hypothesis testing, the test statistic (e.g. *F*, *t*, *r*) with confidence intervals, effect sizes, degrees of freedom and *P* value noted<br>*Give P values as exact values whenever suitable.*
☒	☐	For Bayesian analysis, information on the choice of priors and Markov chain Monte Carlo settings
☒	☐	For hierarchical and complex designs, identification of the appropriate level for tests and full reporting of outcomes
☒	☐	Estimates of effect sizes (e.g. Cohen's *d*, Pearson's *r*), indicating how they were calculated

Our web collection on statistics for biologists contains articles on many of the points above.

Software and code

Policy information about availability of computer code

Data collection	The H&E and Sirius Red results were collected by NIS-Elements 3.2 and Digital panoramic scanner (non fluorescent) Wisnap WS-10. RNA-seq data were collected by the Illumina Novaseq platform. Metabolite content were collected by a QTRAP 5500 system. ChemiDoc™ MP Imaging System (Catalog Number: 12003154) were used for imaging and analyzing western blots. Other data were collected with Microsoft Excel 2019.
Data analysis	All charts were generated using GraphPad Prism software 9.4.1. SPSS version 23.0 was used for statistical analysis. The sample sizes were determined by power analysis using StatMate version 2.0. The metabolites were quantified using Analyst software version 1.6.3 from AB SCIEX. Gene expression was quantified by featureCounts v2.0.1. All downstream analyses were performed in R v4.2.1. We used the edgeR v3.38.4 R package for differential expression analysis. Gene Ontology (GO) enrichment analysis and transcription factor enrichment analysis were conducted by the clusterProfiler v4.4.4 R package.

For manuscripts utilizing custom algorithms or software that are central to the research but not yet described in published literature, software must be made available to editors and reviewers. We strongly encourage code deposition in a community repository (e.g. GitHub). See the Nature Portfolio guidelines for submitting code & software for further information.

Data

Policy information about availability of data

All manuscripts must include a data availability statement. This statement should provide the following information, where applicable:

- Accession codes, unique identifiers, or web links for publicly available datasets
- A description of any restrictions on data availability
- For clinical datasets or third party data, please ensure that the statement adheres to our policy

All of the data supporting the findings of this study are included in the Article and Supplementary information. The RNA sequencing data generated in this study have been deposited in the GEO database under accession code GSE262135 [https://www.ncbi.nlm.nih.gov/geo/query/acc.cgi?acc=GSE262135]. The data generated in this study are provided in the Supplementary Information/Source Data file. The Single-cell RNA sequencing data used in this study are available in the GEO database under accession code GSE166504 [https://www.ncbi.nlm.nih.gov/geo/query/acc.cgi?acc=GSE166504]. Source data are provided with this paper.

Research involving human participants, their data, or biological material

Policy information about studies with human participants or human data. See also policy information about sex, gender (identity/presentation), and sexual orientation and race, ethnicity and racism.

Reporting on sex and gender	We collected serum samples from both male and female subjects. The information regarding the sex of each participant was collected by self-reporting via a questionnaire.
Reporting on race, ethnicity, or other socially relevant groupings	N/A
Population characteristics	Serum samples were obtained from 16 healthy control donors and 16 individuals with NASH (age span of recruited participants was 21-67 years and with a BMI of 18-38kg/m2). The inclusion criteria were as follows: NASH disease diagnosis was in accordance with the Guidelines of Prevention and Treatment of Non-Alcoholic Fatty Liver Disease: a 2018 Update prepared by the National Workshop on Fatty Liver and Alcoholic Liver Disease, Chinese Society of Hepatology, Chinese Medical Association; Fatty Liver Experts Committee, Chinese Medical Doctor Association. The diagnosis requires the patient to have histological evidence of diffuse hepatocyte steatosis, intrahepatic inflammation and fibrosis, and persistent serum ALT and GGT increases. Patients with alcoholic liver disease, type 3 hepatitis C virus infection, autoimmune hepatitis, hepatolenticular degeneration and drug-induced liver disease were excluded. A FibroScan liver elasticity test was performed to support the diagnosis. All patients were newly diagnosed with NASH and did not receive relevant treatment. Healthy volunteers were also recruited from Peking University People's Hospital. They were required to have normal serum ALT and GGT levels. FibroScan indicated that their liver elasticity was normal. Their age, sex and BMI were matched to those of NASH patients.
Recruitment	16 healthy control donors and 16 individuals with NASH were recruited from Peking University People's Hospital. Although there might be some self-selection bias as participants were eager to take part in the study, all subjects met the predefined objective criteria for inclusion and exclusion to minimize the impacts on the study. All of the participants provided written informed consent.<br>The inclusion criteria were as follows: NASH disease diagnosis was in accordance with the Guidelines of Prevention and Treatment of Non-Alcoholic Fatty Liver Disease: a 2018 Update prepared by the National Workshop on Fatty Liver and Alcoholic Liver Disease, Chinese Society of Hepatology, Chinese Medical Association; Fatty Liver Experts Committee, Chinese Medical Doctor Association. The diagnosis requires the patient to have histological evidence of diffuse hepatocyte steatosis, intrahepatic inflammation and fibrosis, and persistent serum ALT and GGT increases. Patients with alcoholic liver disease, type 3 hepatitis C virus infection, hepatolenticular degeneration and drug-induced liver disease were excluded. A FibroScan liver elasticity test was performed to support the diagnosis. All patients were newly diagnosed with NASH and did not receive relevant treatment. Healthy volunteers were also recruited from Peking University People's Hospital. They were required to have normal serum ALT and GGT levels. FibroScan indicated that their liver elasticity was normal. Their age, sex and BMI were matched to those of NASH patients.
Ethics oversight	With the approval of the Ethics Committee of Peking University People's Hospital (Ethics Review Approval No.: 2021PHB124-001), all volunteers who participated in the study signed a written informed consent form.

Note that full information on the approval of the study protocol must also be provided in the manuscript.

Field-specific reporting

Please select the one below that is the best fit for your research. If you are not sure, read the appropriate sections before making your selection.

☒ Life sciences ☐ Behavioural & social sciences ☐ Ecological, evolutionary & environmental sciences

For a reference copy of the document with all sections, see nature.com/documents/nr-reporting-summary-flat.pdf

Life sciences study design

All studies must disclose on these points even when the disclosure is negative.

Sample size	For animal experiments, there were at least 5 mice per group in order to obtain statistical significance, for in vitro or ex vivo study , we have at least 3 biological replicates, which were similar to those previously used by us in this field (ref. 6). For human subjects, GraphPad StatMate 2.0 software was used for the sample size calculation of serum ALT. Standard deviation was obtained based on the level of serum ALT from preliminary experiment. The software provided an estimate of effect size, and indicated the appropriateness of sample sizes chosen. A sample size of 10 in control group and 10 in NASH group has a 80% power to detect a difference between the two group with a significance level (alpha) of 0.05 (two-tailed). Group sample sizes of 10 and 10 achieve 99.999% power to detect a significant difference of the serum ALT between the two groups.
Data exclusions	No data was excluded.
Replication	All experimental findings were repeated at twice or more times. All attempts at replication were successful.
Randomization	Human subjects were randomly selected under the shown criteria, listed in the Online Methods. Participants diagnosed with NASH according to diagnostic criteria were allocated into the NASH group, while those who have normal serum ALT and GGT levels and normal liver elasticity were allocated to the control group.<br>6-week-old or 8-week-old mice male mice were divided at random into experimental groups, with at least 5 mice per group, and the mice did not show differences before treatment. For sample testing, samples were processed tested in random orders. For cell based assays, the individual treatment wells were randomly assigned.
Blinding	The investigators involved in this study were not completely blinded during data collection and/or analysis during the sample collection and analysis. Histological analysis of the liver were randomized and blindly analyzed by a certified pathologist. Investigators were blinded to group allocation during data collection and analysis. But during the in vivo treatment of animals it was not blinded, as the investigators were required to be aware of the reagents being administered to the mice.

Reporting for specific materials, systems and methods

We require information from authors about some types of materials, experimental systems and methods used in many studies. Here, indicate whether each material, system or method listed is relevant to your study. If you are not sure if a list item applies to your research, read the appropriate section before selecting a response.

Materials & experimental systems

n/a	Involved in the study	
☐	☒	Antibodies
☐	☒	Eukaryotic cell lines
☒	☐	Palaeontology and archaeology
☐	☒	Animals and other organisms
☒	☐	Clinical data
☒	☐	Dual use research of concern
☒	☐	Plants

Methods

n/a	Involved in the study	
☒	☐	ChIP-seq
☐	☒	Flow cytometry
☒	☐	MRI-based neuroimaging

Antibodies

Antibodies used	The following antibodies were used: anti-caspase-1 (1:1000, CST, #24232, Monoclonal), anti-cleaved caspase-1 (1:1000, CST, #89332, Monoclonal), anti-HIF-2α (1:1000, Novus, NB100-132, Clone: ep190b), anti-ARNT (1:1000, Santa Cruz, sc-55526, Monoclonal), anti-GAPDH (1:1000, CST, #5174, Monoclonal) and anti-β-Actin (1:1000, Abclonal, AC038, Clone: ARC5115-01), APC/cy7 anti-CD45 (1:400, BioLegend, 157204, Clone:30-F11), FITC anti-CD11b (1:400, BioLegend, 101205, Clone: M1/70), APC anti-F4/80 (1:400, eBioscience, 17-4801-82, Clone:BM8), anti-HIF-1a (1:400, Proteintech, 20960-1-AP, Polyclonal), anti-HIF-2a PE (1:400, Novus, NB100-122PE, Polyclonal),HRP-conjugated Goat anti-Mouse IgG (H+L) (1:2000, ABclonal, AS003), HRP-conjugated Goat anti-Rabbit IgG (H+L) (1:2000, ABclonal,AS014)
Validation	All antibodies were validated by the supplier (CST, Novus, Santa Cruz, Abclonal, BD, eBioscience) and were checked in the lab by comparing manufacturers or in house results. The validation statement and the relevant citation information is listed in the link:<br>1.anti-caspase-1 (1:1000, CST, #24232, Monoclonal):https://www.cellsignal.cn/products/primary-antibodies/caspase-1-e2z1c-rabbit-mab/24232, Product Citations: 110,Monoclonal antibody is produced by immunizing animals with a synthetic peptide corresponding to residues near the carboxy terminus of mouse caspase-1 protein.Caspase-1 (E2Z1C) Rabbit mAb recognizes endogenous levels of total mouse caspase-1 protein. This antibody detects pro-caspase-1 and the p10 subunit of activated caspase-1.<br>2.anti-cleaved caspase-1 (1:1000, CST, #89332, Monoclonal), https://www.cellsignal.cn/products/primary-antibodies/cleaved-caspase-1-asp296-e2g2i-rabbit-mab/89332,Cleaved Caspase-1 (Asp296) (E2G2I) Rabbit mAb recognizes endogenous levels of caspase-1 protein only when cleaved at Asp296. A non-specific band is detected at 70 kDa in some cells.Monoclonal antibody is produced by immunizing animals with a synthetic peptide corresponding to residues surrounding Asp296 of mouse caspase-1 protein. Product Citations: 153.<br>3. anti-HIF-2α (1:1000, Novus, NB100-132, Clone: ep190b), hhttps://www.novusbio.com/products/hif-2-alpha-epas1-antibody-ep190b_nb100-132,References: Semenza, G. L., Agani, F., Feldser, D., Iyer, N., Kotch, L., Laughner, E., & Yu, A. (2000). Hypoxia, HIF-1,

and the pathophysiology of common human diseases. Advances in Experimental Medicine and Biology.Theoretical MW : 96.5 kDa. Disclaimer note: The observed molecular weight of the protein may vary from the listed predicted molecular weight due to post translational modifications, post translation cleavages, relative charges, and other experimental factors.
4. anti-ARNT (1:1000, Santa Cruz, sc-55526, Monoclonal), https://www.scbt.com/p/arnt-1-antibody-h-10,Arnt 1 Antibody (H-10) is a mouse monoclonal IgG1 κ, cited in 17 publications, provided at 200 µg/ml, 17 citations in various scientific publications.
5. anti-GAPDH (1:1000, CST, #5174, Monoclonal) , https://www.cellsignal.cn/products/primary-antibodies/gapdh-d16h11-xp-rabbit-mab/5174, GAPDH (D16H11) XP® Rabbit mAb detects endogenous levels of total GAPDH protein.
6. anti-β-Actin (1:1000, Abclonal, AC038, Clone:ARC5115-01), https://abclonal.com.cn/catalog/AC038, Affinity purification. Buffer: PBS with 0.05% proclin300,0.05% BSA,50% glycerol,pH7.3.
7. APC/cy7 anti-CD45 (1:400, BioLegend, 157204, Clone:30-F11), https://www.biolegend.com/en-us/products/apc-cyanine7-anti-mouse-cd45-antibody-2530?GroupID=BLG1932, Each lot of this antibody is quality control tested by immunofluorescent staining with flow cytometric analysis. For flow cytometric staining, the suggested use of this reagent is = 0.25 µg per 106 cells in 100 µl volume. It is recommended that the reagent be titrated for optimal performance for each application.
8. FITC anti-CD11b (1:400, BioLegend, 101205, Clone: M1/70), https://www.biolegend.com/en-gb/products/fitc-anti-mouse-human-cd11b-antibody-347,Each lot of this antibody is quality control tested by immunofluorescent staining with flow cytometric analysis. For flow cytometric staining, the suggested use of this reagent is ≤ 0.25 µg per 106 cells in 100 µl volume. It is recommended that the reagent be titrated for optimal performance for each application.
9. APC anti-F4/80 (1:400, eBioscience, 17-4801-82, Clone:BM8), https://www.thermofisher.cn/cn/zh/antibody/product/F4-80-Antibody-clone-BM8-Monoclonal/17-4801-82.The BM8 antibody has been tested by flow cytometric analysis of mouse resident peritoneal exudate cells. This can be used at less than or equal to 2 µg per test. A test is defined as the amount (µg) of antibody that will stain a cell sample in a final volume of 100 µL. Cell number should be determined empirically but can range from 10^5 to 10^8 cells/test. It is recommended that the antibody be carefully titrated for optimal performance in the assay of interest.
10. anti-HIF-1a (1:1000, Proteintech, 20960-1-AP, Polyclonal), https://www.ptgcn.com/Products/HIF1A-Antibody-20960-1-AP.htm,20960-1-AP targets HIF-1 alpha in WB, IP, IHC, IF, FC, CoIP, ChIP, Cell treatment, ELISA applications and shows reactivity with human samples.
11. anti-HIF-2a PE (1:200, Novus, NB100-122PE, Polyclonal), https://www.novusbio.com/products/hif-2-alpha-epas1-antibody_nb100-122pe,Use in Mouse reported in scientific literature (PMID:33758176IF: 7.0 Q2).
12. HRP-conjugated Goat anti-Mouse IgG (H+L) (1:2000, ABclonal, AS003), https://abclonal.com.cn/catalog/AS003
13. HRP-conjugated Goat anti-Rabbit IgG (H+L) (1:2000, ABclonal,AS014),https://abclonal.com.cn/catalog/AS014

Eukaryotic cell lines

Policy information about cell lines and Sex and Gender in Research

Cell line source(s)	The HEK293T and LX-2 cell line used in this study was purchased from the National Collection of Authenticated Cell Cultures.
Authentication	The HEK293T and LX-2 cell line is purchased from the National Collection of Authenticated Cell Cultures, and the cell lines undergo authentication tests during the accessioning process. This process is described in the online website: https://www.cellbank.org.cn/estandard.php. Observations of recovery and growth are recorded along with morphological appearance. Although no detailed authentication information by authors, the strain was used at very low passages in the study and no phenotype was analyzed from the cell line.
Mycoplasma contamination	The Cell lines were tested for mycoplasma contamination, and the cell lines were free of mycoplasma contaminations.
Commonly misidentified lines (See ICLAC register)	No commonly misidentified cell lines were used.

Animals and other research organisms

Policy information about studies involving animals; ARRIVE guidelines recommended for reporting animal research, and Sex and Gender in Research

Laboratory animals	C57BL/6J wild-type male mice were purchased from the Department of Laboratory Animal Science, Peking University Health Science Center. Hif2αfl/fl, Hif2αLSL/LSL and Lyz2-cre mice which carrying Cre recombinase under the control of the Lyz2 promoter were purchased from Jackson Lab. Hif2αfl/fl or Hif2αLSL/LSL mice were bred with Lyz2-cre mice to generate Hif2αΔLysm mice and LysMHif2αLSL/LSL mice.<br>Mice were randomly divided into different groups and raised in cages under standard SPF laboratory conditions with free access to water and feed. The temperature was maintained at 21-24 °C , and the humidity was maintained at 40-70%. The light was on from 08:00 to 20:00. The animal use licence number was SYXK (Beijing) 2011-0039. All animal experiments complied with the rules for the use of experimental animals, treatment and euthanasia approved by Peking University Health Science Center (permit: LA2020481).<br>For the So(d18:1) intraperitoneal injection experiment, the So(d16:1) and So(d18:1) was suspended in 0.5% Carboxymethylcellulose sodium (CMC-Na) solution. 6-week-old male mice were randomly fed the CDAA-HFD for 8 weeks with vehicle (5 % CMC-Na), So(d16:1) (10 mg kg-1 body weight) or So(d18:1) (10 mg/kg body weight) injected intraperitoneally every day. For the FG-4592 (MCE, HY-13426) intraperitoneal injection experiment, 6-week-old male mice were randomly fed the CDAA-HFD for 8 weeks with vehicle or FG-4592 (25 mg/kg body weight) injected intraperitoneally every day. For the Hif2αΔLysm mice and LysMHif2αLSL/LSL mice experiment, 6-week-old male mice were randomly fed the CDAA-HFD for 8 weeks.
Wild animals	No wild animals were used in the study.
Reporting on sex	In epidemiological studies, male sex has consistently shown strong associations with NASH incidence (PMID: 37040843). Male mice were applied in this study.

Field-collected samples | No field collected animals were used in the study.

Ethics oversight | All animal experimental procedures were approved by the Animal Care and Use Committee of Peking University Health Science Center according to the national legislation for animal care(permit: LA2020481).

Note that full information on the approval of the study protocol must also be provided in the manuscript.

Flow Cytometry

Plots

Confirm that:

☒ The axis labels state the marker and fluorochrome used (e.g. CD4-FITC).

☒ The axis scales are clearly visible. Include numbers along axes only for bottom left plot of group (a 'group' is an analysis of identical markers).

☒ All plots are contour plots with outliers or pseudocolor plots.

☒ A numerical value for number of cells or percentage (with statistics) is provided.

Methodology

Sample preparation	Isolated liver nonparenchymal cells were washed in PBS buffer containing 10% FBS, and red cells were removed. The cells were stained with specific antibodies at 4 °C for 30 minutes protected from light, washed with cold PBS 3 times, and analysed by flow cytometry using FACSCelesta (BD).
Instrument	FACSCelesta (BD).
Software	FlowJo software (TreeStar).
Cell population abundance	At least 30000 cells were acquired for each samples.
Gating strategy	Gating strategy for macrophages: Cells were activated and gated on FSC vs SSC, and further gated on live cells. Live cells were further gated on CD45+ cells in which F4/80+CD11b+ were tested.

☒ Tick this box to confirm that a figure exemplifying the gating strategy is provided in the Supplementary Information.
