# Supplementary figures and images for: Sphingosine d18:1 promotes nonalcoholic steatohepatitis by inhibiting macrophage HIF-2α
Source: Nat Commun. 2024 Jun 4;15:4755. doi: 10.1038/s41467-024-48954-2 (PMC11150497; doi:10.1038/s41467-024-48954-2)

**a**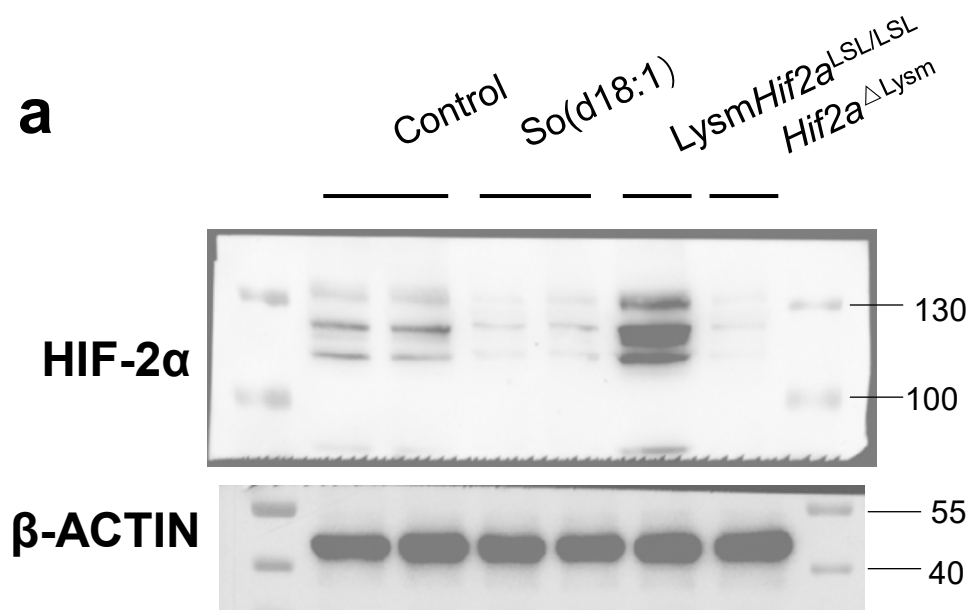

( Refer to fig 3F)

**b**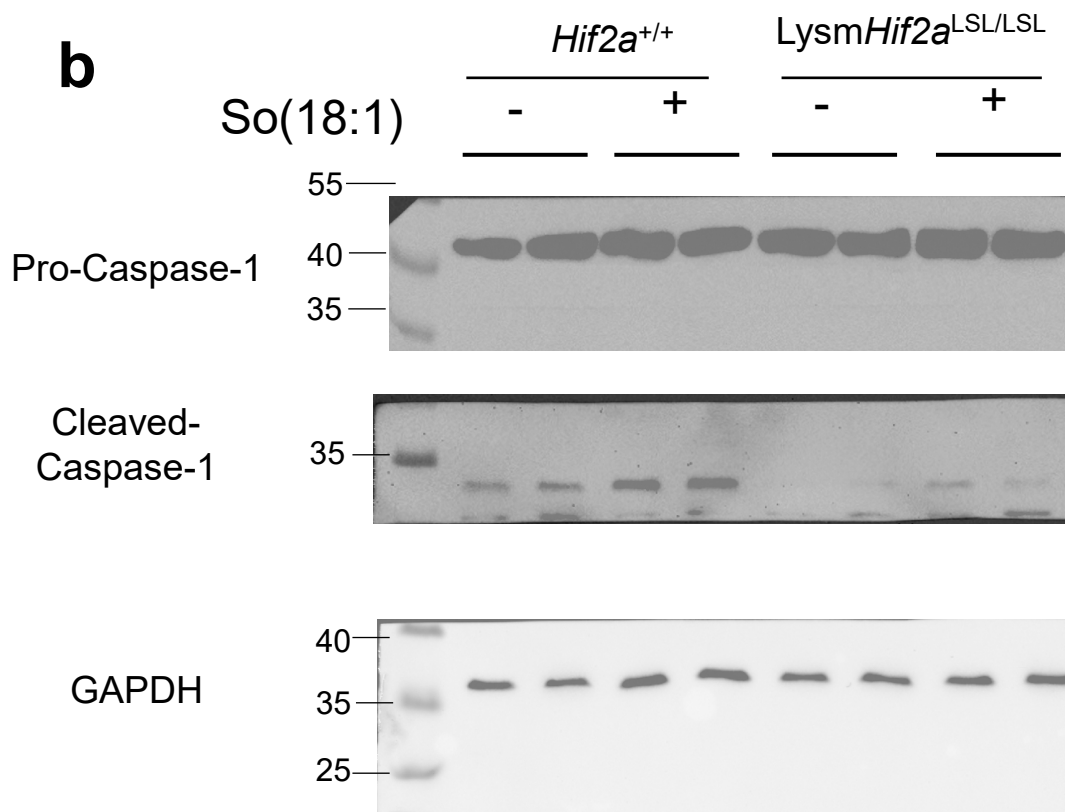

( Refer to fig 3G)

**C**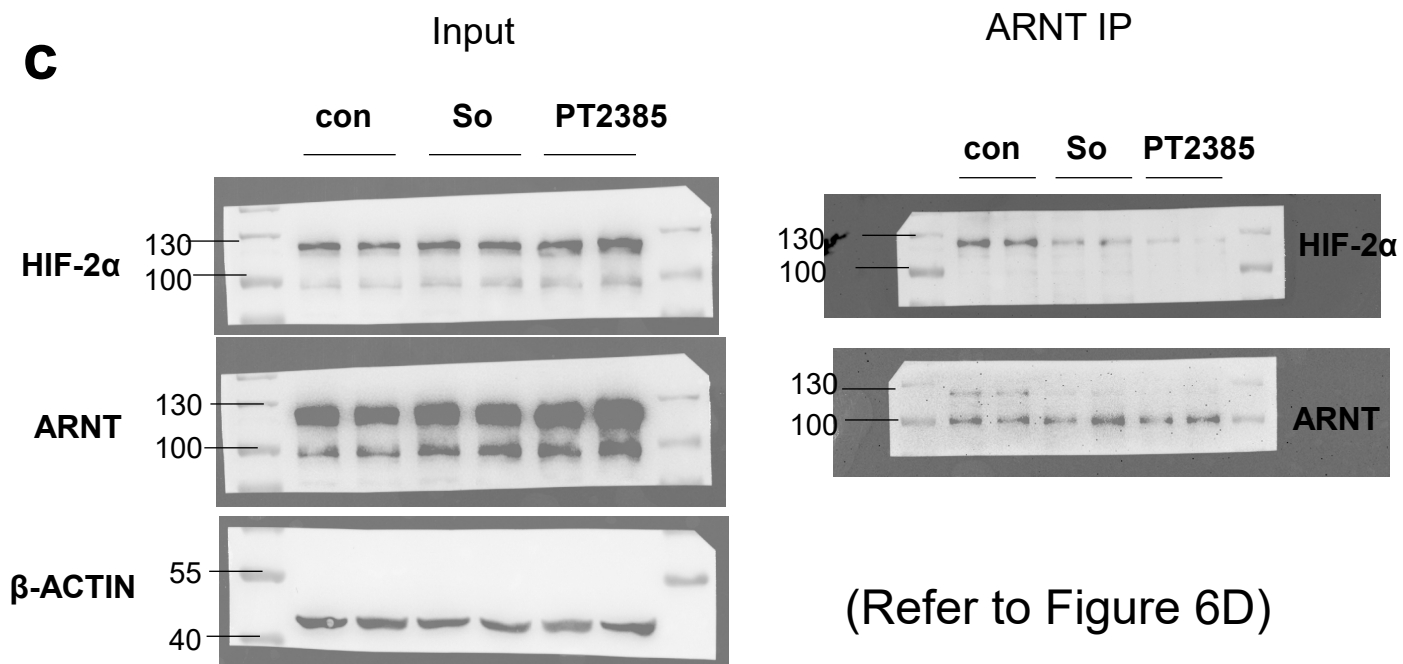**d**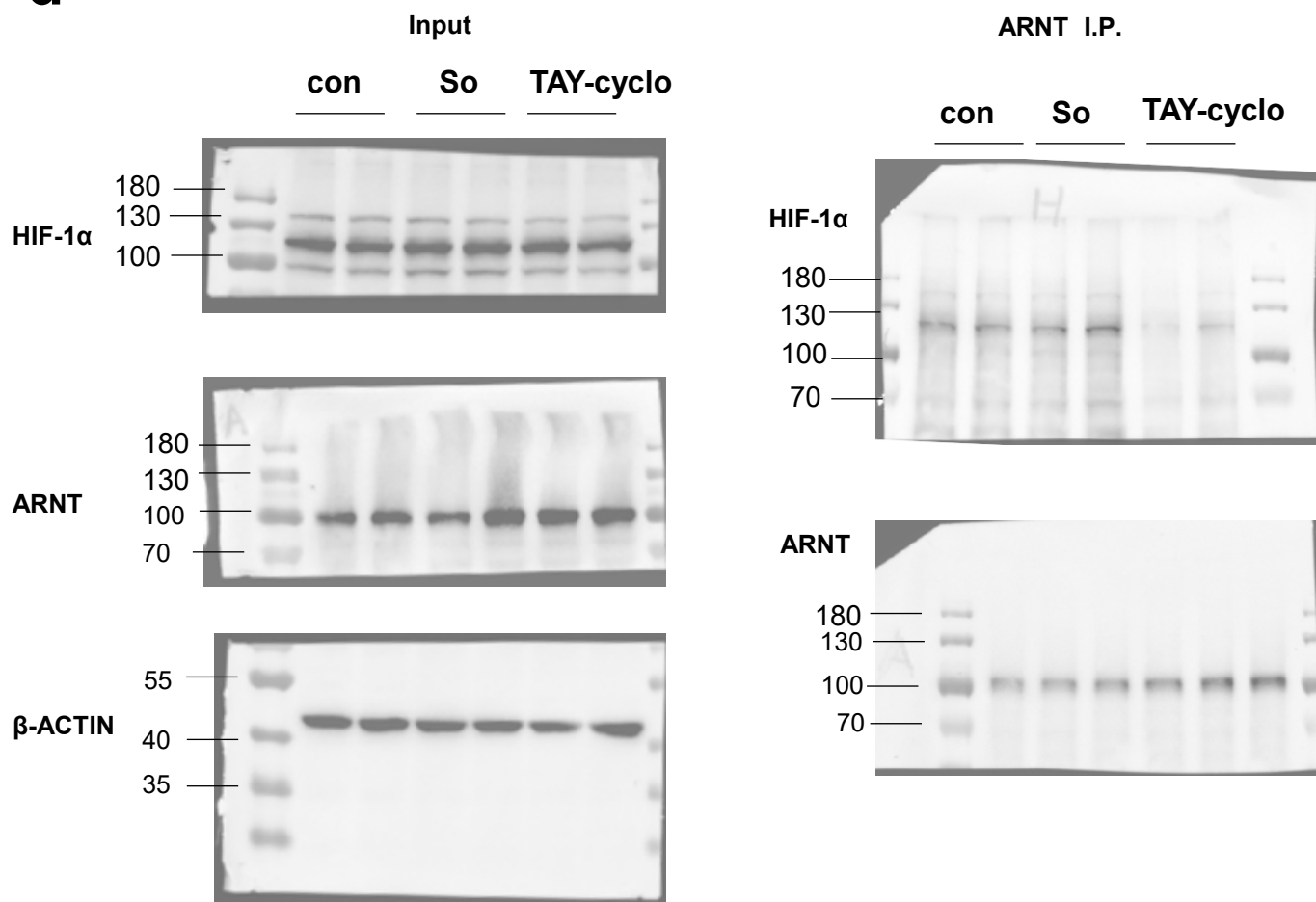

Supplement: Supplementary file 4 — Source Data [file 41467_2024_48954_MOESM4_ESM.zip › 5-source data-20240511/uncropped blots.pdf]
